# Physiology of a Hybrid Pathway for Nicotine Catabolism in Bacteria

**DOI:** 10.3389/fmicb.2020.598207

**Published:** 2020-11-12

**Authors:** Haiyan Huang, Jinmeng Shang, Shuning Wang

**Affiliations:** ^1^State Key Laboratory of Microbial Technology, Microbial Technology Institute, Shandong University, Qingdao, China; ^2^Institute of Basic Medicine, Shandong First Medical University & Shandong Academy of Medical Science, Jinan, China

**Keywords:** nicotine, degrading pathway, enzymes, gene cluster, catabolism, functionalized pyridines

## Abstract

Nicotine is a major *N*-heterocyclic aromatic alkaloid produced in tobacco plants and the main toxic chemical in tobacco waste. Due to its complex physiological effects and toxicity, it has become a concern both in terms of public health and the environment. A number of bacteria belonging to the genera *Arthrobacter* and *Pseudomonas* can degrade nicotine *via* the pyridine and pyrrollidine pathways. Recently, a novel hybrid of the pyridine and pyrrolidine pathways (also known as the VPP pathway) was found in the *Rhizobiale* group bacteria *Agrobacterium tumefaciens* S33, *Shinella* sp. HZN7 and *Ochrobactrum* sp. SJY1 as well as in other group bacteria. The special mosaic pathway has attracted much attention from microbiologists in terms of the study of their molecular and biochemical mechanisms. This will benefit the development of new biotechnologies in terms of the use of nicotine, the enzymes involved in its catabolism, and the microorganisms capable of degrading the alkaloid. In this pathway, some metabolites are hydroxylated in the pyridine ring or modified in the side chain with active groups, which can be used as precursors for the synthesis of some important compounds in the pharmaceutical and agricultural industries. Moreover, some enzymes may be used for industrial biocatalysis to transform pyridine derivatives into desired chemicals. Here, we review the molecular and biochemical basis of the hybrid nicotine-degrading pathway and discuss the electron transport in its oxidative degradation for energy conservation and bacterial growth.

## Introduction

Nicotine is the main alkaloid produced in tobacco (Bush et al., 1999). In addition to the central nervous system, it has complex physiological effects on humans, causing people to get addicted to tobacco and develop various smoking-related diseases (Hecht, 1999; Campain, 2004; Benowitz, 2010). Due to its toxicity, it is also an environmental concern when considering the smoke and the waste accumulation during tobacco consumption and manufacturing (Novotny and Zhao, 1999). Its production and consumption are thus regulated according to the World Health Organization Framework Convention on Tobacco Control. For centuries, however, tobacco has been widely planted as an important economic nonfood crop around the world. It is therefore imperative to develop alternative technologies to utilize tobacco and dispose of waste. Nicotine has attracted much interest as a substrate to synthesize or biotransform into renewable functionalized pyridines, such as drugs and insecticides, as well as a precursor for synthesis of these molecules (Wang et al., 2012). For example, some nicotine-degrading bacteria can specifically hydroxylate the pyridine ring of nicotine at the 6-position or 2- and 5-positions, which is a difficult procedure when using chemical methods (Schmid et al., 2001; Wang et al., 2005; Ondachi and Comins, 2010). These bacteria or their enzymes involved in nicotine catabolism, could consequently be used as biocatalysts in the processes.

**Figure 1:**
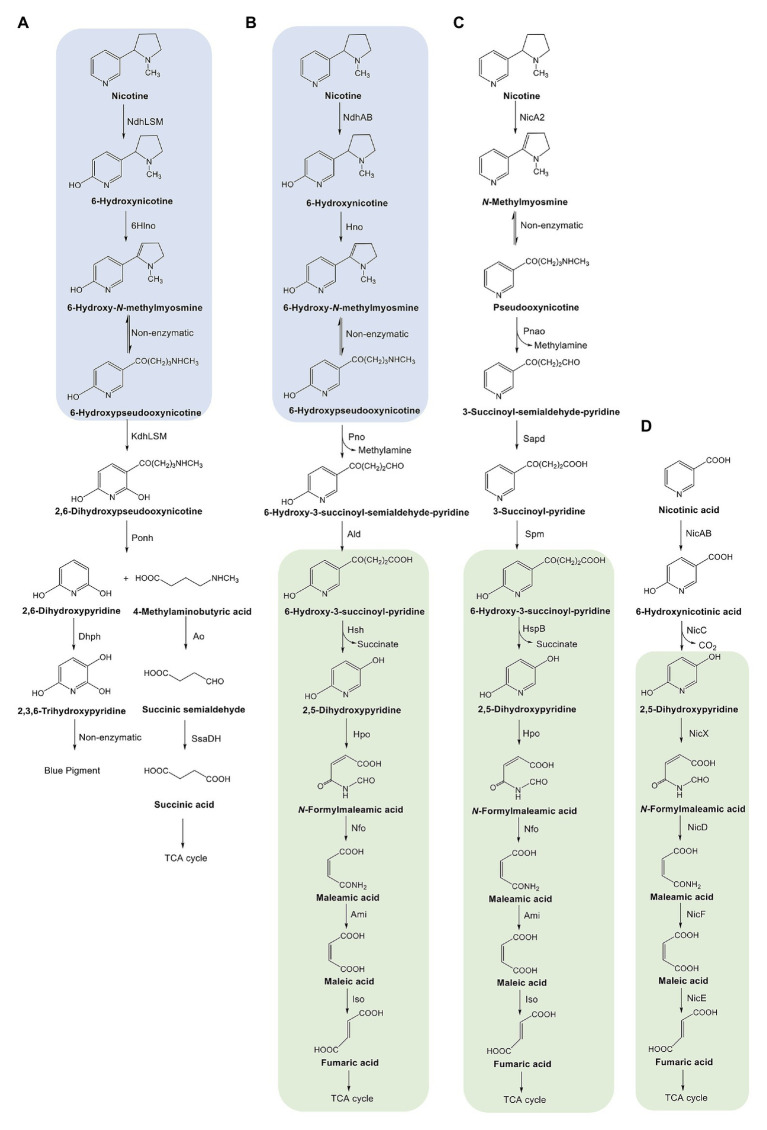
Scheme depicting the pathways for degrading nicotine and nicotinate in bacteria. **(A)** Pyridine pathway of nicotine degradation in *Arthrobacter*. Ndh, nicotine dehydrogenase; 6Hlno, 6-hydroxy-L-nicotine oxidase; Kdh, ketone dehydrogenase; Ponh, 2,6-dihydroxypseudooxynicotine hydrolase; Dhph, 2,6-dihydroxypyridine 3-hydroxylase. **(B)** A hybrid of pyridine and pyrrolidine pathways for nicotine degradation discussed in this review. See Figure 2 legend for enzyme abbreviations. **(C)** Pyrrolidine pathway of nicotine degradation in *Pseudomonas*. NicA, nicotine oxidoreductase; Pnao, pseudooxynicotine amine oxidase; Sapd, 3-succinoylsemialdehyde-pyridne dehydrogenase; SpmABC, 3-succinoylpyridine monooxygenase; HspB, 6-hydroxy-3-succinoylpyridine hydroxylase; Hpo, 2,5-dihydroxypyridine dioxygenase; Nfo, *N*-formylmaleamate deformylase; Ami, maleamate amidohydrolase; Iso, maleate *cis*/*trans*-isomerase. **(D)** Nicotinate-degrading pathway in *Pseudomonas*. NicAB, nicotinate hydroxylase; NicC, 6-hydroxynicotinate monooxygenase; NicX, 2,5-dihydroxypyridine dioxygenase; NicD, *N*-formylmaleamate deformylase; NicF, maleamate amidohydrolase; NicE, maleate *cis*/*trans*-isomerase. The parts shaded in blue indicate the same steps in the pyridine and hybrid pathways; those shaded in green indicate the same steps in the hybrid, pyrrolidine, and nicotinate-degrading pathways. The figure is modified from Huang et al., 2017, permitted by a Creative Commons Attribution 4.0 International License (http://creativecommons.org/licenses/by/4.0/).

Many microorganisms can degrade nicotine *via* various biochemical pathways, including the demethylation pathway in *Aspergillus oryzae* (Meng et al., 2010), the pyridine pathway in *Arthrobacter* (Brandsch, 2006; Figure 1A), and the pyrrolidine pathway in *Pseudomonas* (Wang et al., 2007; Tang et al., 2012, 2013; Figure 1C). The molecular mechanisms underlying the pyridine and pyrrolidine pathways in bacteria have been well investigated. Recently, a novel hybrid of the pyridine and pyrrolidine pathways (also called the VPP pathway) was discovered in the *Rhizobiale* group bacterium *Agrobacterium tumefaciens* S33 (Wang et al., 2012; Figure 1B) and was later also found in *Shinella* sp. HZN7 (Ma et al., 2014), *Ochrobactrum* sp. SJY1 (Yu et al., 2015), and other group bacteria, such as *Sphingomonas melonis* TY (Wang et al., 2017) and *Pseudomonas geniculata* N1 (Wang et al., 2019b). Here, we summarize the progress in molecular and biochemical studies on the hybrid pathway, considering its unique mosaic feature and valuable functionalized pyridine intermediates. Although most core genes involved in the pathway have been identified in these bacteria, the enzymatic properties and kinetics are mainly determined for those from the three *Rhizobiale* group bacteria, which are the main focus of this mini review; for further information, the reader is referred to a recent review paper (Mu et al., 2020).

## A Hybrid of the Pyridine and Pyrrolidine Pathways for Nicotine Degradation in Bacteria

The bacterial catabolism of nicotine includes two crucial actions: hydroxylation of the pyridine ring and dehydrogenation of the pyrrolidine ring of nicotine. Different pathways follow varying sequences. The pyridine pathway (Figure 1A) starts by attacking the pyridine ring to hydroxylate nicotine into 6-hydroxynicotine. The product is then dehydrogenated in the pyrrolidine ring to form 6-hydroxy-*N*-methylmyosmine, which is then spontaneously hydrolyzed to break the pyrrolidine ring and produce 6-hydroxy-pseudooxynicotine. In contrast, the pyrrolidine pathway (Figure 1C) starts by dehydrogenating the pyrrolidine ring into *N*-methylmyosmine, which is then spontaneously hydrolyzed to pseudooxynicotine. After oxidation of pseudooxynicotine to 3-succinoyl-semialdehyde-pyridine and further dehydrogenation to 3-succinoyl-pyridine, the pyridine ring is hydroxylated to produce 6-hydroxy-3-succinoyl-pyridine. The hybrid pathway (Figure 1B) conforms with the pyridine pathway in the steps from nicotine to 6-hydroxy-pseudooxynicotine. However, the oxidation of 6-hydroxy-pseudooxynicotine to 6-hydroxy-3-succinoyl-pyridine *via* its semialdehyde intermediate in the hybrid is similar to that of the non-hydroxylated intermediates in the pyrrolidine pathway. The first common intermediate of the hybrid and the pyrrolidine pathways is 6-hydroxy-3-succinoyl-pyridine. The next steps for the breakdown of the side chain of 6-hydroxy-3-succinoyl-pyridine and decomposition of the pyridine ring in the hybrid are identical to those in the pyrrolidine pathway. Interestingly, the steps for 2,5-dihydroxypyridine degradation are also identical to those in the nicotinate-degrading pathway (Figure 1D).

Among the intermediates of the hybrid pathway, several are hydroxylated in the pyridine ring or modified in the side chain with active groups, which can be used as precursors for the synthesis of some compounds of pharmaceutical and agricultural importance combined with chemical methods (Roduit et al., 1997; Nakano et al., 1999; Wang et al., 2005). For example, 6-hydroxynicotine and 6-hydroxy-3-succinoylpyridine can be chemically transformed into 2,5- or 3,5-disubstituted pyridines, such as the insecticide Imidacloprid, the anti-Parkinson agent SIB-1508Y, and the analgesic epibatidine. The disubstituted pyridine 2,5-dihydroxypyridine can be chemically catalyzed into 5-aminolevulinic acid, which is applied as a universal precursor for porphyrins, herbicides, and plant growth hormones as well as cancer therapy. This provides opportunities to develop new technologies for utilizing tobacco and nicotine.

## The Molecular Basis for the Hybrid Pathway

**Figure 2:**
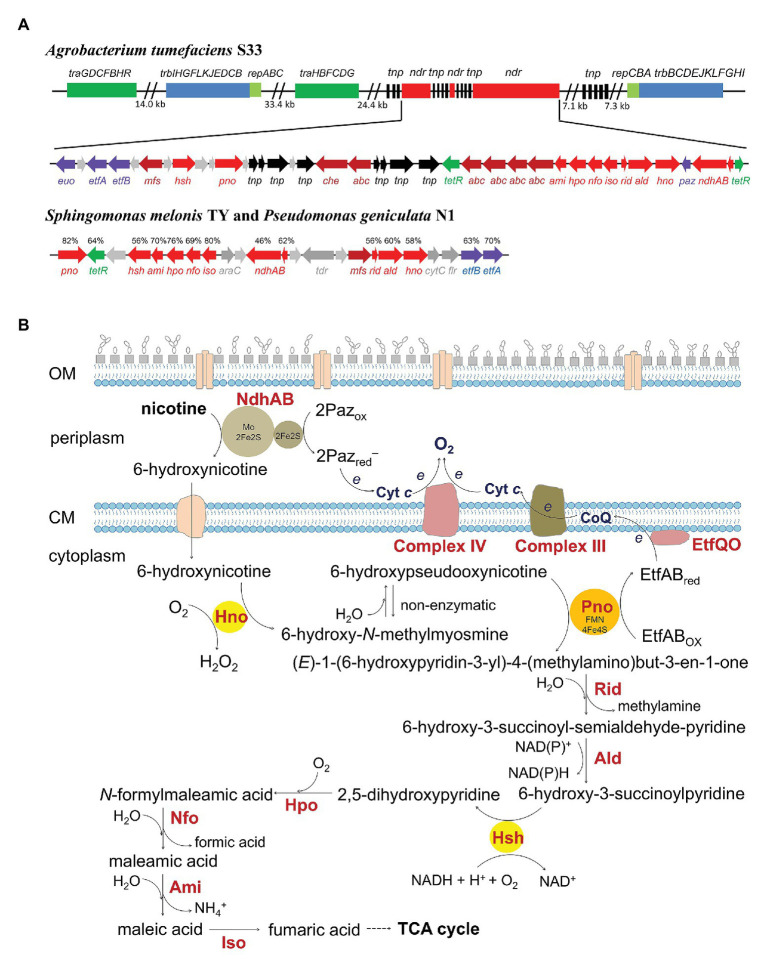
The genetic organization of the gene cluster involved in the hybrid nicotine-degrading pathway in *A. tumefaciens* S33, *S. melonis* TY, and *P. geniculata* N1 **(A)** and the proposed biochemical process of the hybrid for nicotine catabolism **(B)**. *traGDCFBHR* and *trbIHGFLKJEDCB*, conjugal transfer proteins; *repABC*, plasmid partitioning and replication initiation proteins; *ndr*, nicotine-degrading gene cluster; *tnp*, transposase; *euo*, electron transfer flavoprotein:ubiquinone oxidoreductase (EtfQO); *etfAB*, electron transfer flavoprotein; *mfs*, major facilitator superfamily transporter; *hsh*, 6-hydroxy-3-succinoyl-pyridine hydroxylase; *pno*, 6-hydroxypseudooxynicotine dehydrogenase; *che*, chemotaxis protein; *abc*, ABC transporter; *tetR*, TetR family transcriptional regulator; *ami*, maleamate amidohydrolase; *hpo*, 2,5-dihydroxypyridine dioxygenase; *nfo*, *N*-formylmaleamate deformylase; *iso*, maleate cis/trans-isomerase; *rid*, Rid family protein; *ald*, aldehyde dehydrogenase; *hno*, 6-hydroxynicotine oxidase; *paz*, pseudoazurin; *ndhAB*, nicotine dehydrogenase; *araC*, helix-turn-helix (HTH) transcriptional regulator containing an AraC family HTH domain; *tdr*, TonB-dependent receptor; *cytC*, cytochrome *c*; *flr*, flavin reductase. The protein sequence identities compared with those from S33 are shown above the genes. The genetic organization in strain S33 in **(A)** is modified from Huang et al., 2017, permitted by a Creative Commons Attribution 4.0 International License (http://creativecommons.org/licenses/by/4.0/).

Most of the genes responsible for the hybrid pathway in *A. tumefaciens* S33, *Shinella* sp. HZN7, and *Ochrobactrum* sp. SJY1 have been identified by genomic, transcriptomic, and biochemical analyses and gene disruption experiments (Yu et al., 2015, 2016; Qiu et al., 2016; Huang et al., 2017). They form a large cluster located on a genomic island in the circular chromosome of strain S33 (Figure 2A), which also harbors a second linear chromosome (Huang et al., 2017). In contrast, the nicotine-degrading gene cluster in *Shinella* sp. HZN7 is on 155-kb plasmid pShin-05, which harbors one circular chromosome and 12 plasmids. For *Ochrobactrum* sp. SJY1, the location of the nicotine-degrading gene cluster is not clear because only the whole genome shotgun sequence is available. Despite these differences, the arrangement of the gene clusters in the three strains is highly similar (Figure 2A). They are spaced by several genes annotated to encode transposase, group II intron reverse transcriptase/maturase, and DDE endonuclease. In addition, the upstream and/or downstream regions of the gene cluster in S33 and HZN7 contain several gene modules encoding mobile genetic elements for transposition (for example, intergrase and tranposases), conjugal transfer (TraGDCFBHR and TrbIHGFLKJEDCB), and plasmid partition and replication initiation (RepABC), indicating that the nicotine-degrading genes evolved *via* complicated recombination and integration through transposition and conjunction. However, there is no gene for conjugal transfer in strain SJY1. Moreover, transcriptional analyses indicate that these genes are nicotine-inducible, and some genes are co-transcribed and can form single contiguous transcripts (Yu et al., 2015; Huang et al., 2017).

The core nicotine-degrading genes are highly conserved in the three strains, and their encoding proteins are almost identical. Most functional genes and their encoding enzymes have been characterized (see next section). There are still some genes whose functions are only predicted by annotation, including the genes for one major facilitator superfamily transporter (MFS), one chemotaxis protein (Che), five putrescine/spermidine ABC transporters (ABC), and two TetR family transcriptional regulators (TetR). They are predicted to function in substrate sensing and transport as well as transcriptional regulation in nicotine catabolism.

For the cases of *S. melonis* TY and *P. geniculata* N1 (Figure 2A), the organization, arrangement, and sequence of their nicotine-degrading genes is highly identical, which is different from the case of the three *Rhizobiale* species (Wang et al., 2017, 2019b). They are colocalized and tightly clustered without interruption by any mobile genetic element. The nicotine-degrading enzymes show 56–75% and 46–82% identity in DNA sequence and protein sequence, respectively, to those of *Rhizobiale* species. In addition, there are two genes for TetR transcriptional regulator and helix-turn-helix transcriptional regulator, which contains an AraC family HTH domain, in the gene cluster, and there are no genes for the ABC transporter, Che, Euo (electron transfer flavoprotein:ubiquinone oxidoreductase), or Paz (pseudoazurin). The MFS shows no significant similarity to the proteins of *Rhizobiale* species.

Alignment of the protein sequences of the enzymes with the corresponding enzymes involved in the pyridine and pyrrolidine pathways shows that the first three enzymes in the hybrid exhibit low identity to those in the pyridine pathway though they catalyze the same reactions (see the next section; Huang et al., 2017). In contrast, the enzymes for the late steps from 6-hydroxy-3-succinoylpyridine to fumaric acid in the hybrid are highly similar to those of the pyrrolidine pathway and the nicotinate-degrading pathway. This indicates that the enzymes for the starting steps of the hybrid evolve independently from the pyridine pathway, while others for the late steps are closely related to the enzymes in the pyrrolidine pathway and the nicotinate-degrading pathway in molecular evolution. The evolution of the hybrid pathway is therefore not a simple fusion of the genes involved in the pyridine and pyrrolidine pathways but a result of multiple complicated later gene transfers.

## The Biochemical Basis for the Hybrid Pathway

In the hybrid pathway, nicotine is catabolized into fumaric acid *via* 10 steps, which then enters the TCA cycle. Nine core enzymes are responsible for the catalytic process, and the third step is a non-enzymatic reaction (Figure 2B). Among them, six are oxidoreductase, two are hydrolase, and one is an isomerase. In *A. tumefaciens* S33, nicotine dehydrogenase NdhAB, which catalyzes the initial step of hydroxylation of nicotine at the 6-position carbon atom of the pyridine ring, is located in the periplasm of cells, although it was co-purified with 6-hydroxy-pseudooxynicotine dehydrogenase (Pno) at the beginning because of their strong nonspecific interactions (Li et al., 2016; Yu et al., 2017a). Other enzymes in the pathway are found in the cytoplasm. There is a typical twin-arginine translocation system (Tat) signal peptide at the N-terminus of NdhA, which transports NdhAB into the periplasm after synthesis in the cytoplasm. The two-component enzyme harbors a molybdopterin cofactor on NdhA (82.4 kDa) and two [2Fe2S] clusters on NdhB (17.1 kDa), which is similar to the corresponding subunits of the classical three-component molybdopterin-containing hydroxylases belonging to the xanthine oxidase family such as nicotine dehydrogenase NdhLMS from *A. nicotinovorans*. However, NdhAB lacks a FAD-binding subunit, the middle size subunit of the classical hydroxylase. These cofactors of hydroxylase play important roles in transporting electrons from the reducing substrate to the electron acceptor. The typical hydroxylase usually uses NAD(P)^+^ or O_2_ as an electron acceptor, which reacts at the FAD site, and the electrons transfer between molybdopterin and FAD *via* the intervening [2Fe2S] clusters. Due to the lack of FAD, NdhAB cannot use NAD(P)^+^ or O_2_ but utilizes pseudoazurin (Paz) as its physiological electron acceptor. Paz is a 17-kDa blue copper protein and is also transported into the periplasm with the guidance of its special signal peptide. This is consistent with the fact that the gene *paz* is adjacent to *ndhAB* and that its transcriptional level is significantly upregulated when strain S33 grew on nicotine, which is similar to other nicotine-degrading genes. In the presence of Paz, NdhAB presents an activity of 110.5 U/mg at pH 7.0 and 30°C. The apparent *K_m_* values for nicotine and Paz are 1.6 and 3.6 μM, respectively. The ^18^O-labeled experiments indicate that the oxygen atom in the hydroxyl group of the product 6-hydroxynicotine is derived from H_2_O.

The oxidation of 6-hydroxynicotine is catalyzed by 6-hydroxynicotine oxidase (Hno) in the cytoplasm of cells (Yu et al., 2017a,b). The substrate 6-hydroxynicotine must be transported into the cytoplasm before oxidation. The predicted chemotaxis protein (Che), ABC transporter (Abc), and major facilitator superfamily transporter (Mfs) may be responsible for its sensing and transport. Their genes are clustered with the core nicotine-degrading genes and have been verified to be involved in nicotine degradation by transcriptomic analysis (Huang et al., 2017). Hno (48.7 kDa) binds an FAD with a Rossmann-like domain, and converts 6-hydroxynicotine into 6-hydroxy-*N*-methylmyosmine in the presence of O_2_ with the formation of H_2_O_2_ like other flavin-containing amine oxidases. Hno has a 38.9% identity to nicotine oxidase Nox from *Pseudomonas* sp. HZN6, 38.4% to nicotine oxidoreductase NicA2 from *P. putida* S16, and 24.8% to 6-hydroxy-L-nicotine oxidase from *A. nicotinovorans*. It presents a specific activity of 26.4 U/mg at 37°C and pH 9.2 with an apparent *K_m_* of 0.067 mM for 6-hydroxynicotine. The product 6-hydroxy-*N*-methylmyosmine is spontaneously hydrolyzed into 6-hydroxy-pseudooxynicotine. The pyrrolidine ring of nicotine is consequently opened.

The intermediate 6-hydroxy-pseudooxynicotine is further dehydrogenated into 6-hydroxy-3-succinoyl-semialdehyde-pyridine by 6-hydroxy-pseudooxynicotine dehydrogenase (Pno), which was formerly thought to be an oxidase since it catalyzes a reaction similar to that by pseudooxynicotine amine oxidase (Pao or Pnao) from *Pseudomonas* sp. HZN6 and *P. putida* S16 (Li et al., 2016; Wang et al., 2019a). By coupling with the reaction of 6-hydroxynicotine oxidation by Hno, the activity of Pno at 32.3 U/mg and an apparent *K_m_* of 0.37 mM for 6-hydroxy-pseudooxynicotine were estimated at pH 8.5 and 30°C. Because 6-hydroxy-pseudooxynicotine is not commercially available, its analog pseudooxynicotine was used as the substrate to characterize the enzyme. It presents a similar activity (52.8 U/mg) and apparent *K_m_* (0.42 mM) for pseudooxynicotine. In the assays, DCPIP serves as an artificial electron acceptor since NAD(P)^+^, O_2_, or ferredoxin does not function. Pno is a 73-kDa protein harboring an FMN, a [4Fe4S]-cluster, and an ADP. It shows almost no identity to Pao/Pano (less than 12%), but exhibits high identity to histamine dehydrogenase (48%) from *Pimelobacter simplex*, trimethylamine dehydrogenase (40%) from *Methylophilus methylotrophus* W3A1, and dimethylamine dehydrogenase (39%) from *Hyphomicrobium* sp. strain X. Recently, the crystal structure of the enzyme from *P. geniculata* N1 (82% identity to Pno from strain S33) was determined (Liu et al., 2020). It shows that FMN is not covalently bound to the protein and the isoalloxazine ring is planar, which is distinct from the case of trimethylamine dehydrogenase and histamine dehydrogenase. Differently, the enzyme from *P. geniculata* N1 is found to catalyze 6-hydroxy-pseudooxynicotine to 6-hydroxy-3-succinoyl-semialdehyde-pyridine and 6-hydroxy-3-succinoyl-pyridine with a very low activity (Wang et al., 2019b). Pno from strain S33 utilizes electron transfer flavoprotein (EtfAB) as the physiological electron acceptor, which is similar to trimethylamine dehydrogenase and dimethylamine dehydrogenase. The reduced EtfAB can be reoxidized by electron transfer flavoprotein:ubiquinone oxidoreductase (Euo) with CoQ as the electron acceptor. The *etfAB* and *euo* genes are located in the nicotine-degrading gene cluster, and their transcriptional levels are also up-regulated in the presence of nicotine. The ^18^O-labeling experiments showed that the oxygen atom in the aldehyde group of the product 3-succinoyl-semialdehyde-pyridine was derived not from O_2_ but from H_2_O. This evidence indicates that Pno is a new member of EC 1.5.8., which is a group of dehydrogenases acting on the CH-NH group of electron donors with a flavin (EtfAB) as an electron acceptor. Considering the role of Pno *in vivo*, it is assumed that 6-hydroxy-pseudooxynicotine is dehydrogenated to an enamine intermediate [(*E*)-1-(6-hydroxypyridin-3-yl)-4-(methylamino)but-3-en-1-one] by Pno, which is further hydrolyzed to form the deamination product 6-hydroxy-3-succinoyl-semialdehyde-pyridine and methylamine.

It is known that enamine is a highly reactive and unstable compound, which can be isomerized to an imine (Borchert et al., 2019). The two intermediates usually can be spontaneously hydrolyzed into a harmless ketoacid, but this reaction is very slow because of the shortage of free water molecules *in vivo*, which causes their accumulation in cells. This will lead to metabolic damage to the cells because they can covalently bind to pyridoxal 5′-phosphate (PLP) to inactivate PLP-dependent enzymes. RidA protein has been found to preempt the damage of enamine compounds by hydrolyzing them *via* its deaminase activity (Lambrecht et al., 2013). In the nicotine-degrading gene cluster of strain S33, an ORF is annotated to encode a Rid6 subfamily protein, whose transcriptional level is also up-regulated in the presence of nicotine. We predict that Rid6 is required to remove the toxic enamine/imine intermediate produced in the Pno reaction *in vivo*.

In the next step of the reaction, 6-hydroxy-3-succinoyl-semialdehyde-pyridine is oxidized to 6-hydroxy-3-succinoyl-pyridine by a predicted NAD(P)-dependent aldehyde dehydrogenase (Ald), which is encoded by an ORF adjacent to the *hno* gene in the nicotine-degrading gene cluster (Huang et al., 2017). Like other genes in the cluster, its transcriptional level presents a similar up-regulated pattern with nicotine. The protein (49.9 kDa) has 35% identity to the NADP-dependent 3-succinoyl-semialdehyde-pyridine dehydrogenase (Sapd) from *Pseudomonas* sp. HZN6 (Qiu et al., 2012). Its detailed biochemical properties require further characterization.

The sixth step is oxidative decarboxylation of 6-hydroxy-3-succinoyl-pyridine to 2,5-dihydroxypyridine and succinic acid by 6-hydroxy-3-succinoyl-pyridine hydroxylase (Hsh; Li et al., 2014). Hsh is a 90 kDa homodimeric FAD-containing monooxygenase, which exhibits 62.8% identity to with HSP hydroxylase (HspB) in the pyrrolidine pathway of *P. putida* S16. The enzyme also presents a high identity to *p*-nitrophenol monooxygenase from *Pseudomonas* sp. WBC-3 (38%) and *Pseudomonas* sp. NyZ402 (37%), but low identification (approximately 17%) with 6-hydroxynicotinate monooxygenase from *P. putida* KT2440. The protein has two conserved Rossmann fold motifs, which each bind FAD and NADH. However, FAD is easily disassociated from the enzyme during purification. The purified Hsh demonstrates a specific activity of 18.8 U/mg, an apparent *K_m_* of 0.15 mM for 6-hydroxy-3-succinoyl-pyridine, and an apparent *K_m_* of 0.1 mM for NADH at pH 8.0 and 30°C. The enzyme can also use NADPH to substitute NADH in the reaction with a specific activity of 12.9 U/mg and an apparent *K_m_* of 0.35 mM. ^18^O-labeling experiments for HspB from *P. putida* S16 showed that the oxygen atom incorporated into the product 2,5-dihydroxypyrdine is derived from O_2_, while the oxygen introduced into the product succinic acid is from H_2_O (Tang et al., 2011; Yu et al., 2014). Since the second oxygen atom from O_2_ is finally reduced to H_2_O, H_2_O is both a substrate and product in the reaction. Rapid reaction kinetic experiments have shown that HspB is first reduced on its flavin by NADH, then oxidized by O_2_ to form a C_(4a)_-hydroperoxyflavin intermediate, which further reacts with the substrate 6-hydroxy-3-succinoyl-pyridine to form 2,5-dihydroxypyrdine and succinic acid (Yu et al., 2014).

The four enzymes responsible for the last steps of conversion from 2,5-dihydroxypyrdine to fumaric acid in the hybrid pathway are very close to those in the pyrrolidine pathway in *P. putida* S16 and the nicotinate-degrading pathway in *P. putida* KT2440 (Jiménez et al., 2008; Tang et al., 2012; Yao et al., 2013; Huang et al., 2017). The oxidation of 2,5-dihydroxypyridine to break the pyridine ring into *N*-formylmaleamic acid is catalyzed by 2,5-dihydroxypyridine dioxygenase (Hpo, 37.6 kDa for monomer), which exhibits 81% identity to the enzyme from *P. putida* S16 and 42.9% to NicX from *P. putida* KT2440. The enzyme from strain S16 is an Fe(II)-dependent homotrimer and presents a specific activity of 20.5 U/mg and an apparent *K_m_* of 0.14 uM for 2,5-dihydroxypyridine in the presence of Fe^2+^ at pH 7.5 and 20°C (Tang et al., 2012; Yao et al., 2013)*. N*-formylmaleamate deformylase (Nfo, 28.1 kDa) catalyzes the hydrolysis of *N*-formylmaleamic acid into maleamic and formic acids. The enzyme exhibits an identity of 62.3% to Nfo from strain S16 and 58.2% identity to NicD from strain KT2440. Nfo from strain S16 shows a specific activity of 1.1 U/mg and an apparent *K_m_* of 0.94 mM for *N*-formylmaleamic acid at pH 6.7 and 20°C. Next the hydrolysis of maleamic acid into maleic acid and ammonia is catalyzed by maleamate amidohydrolase (Ami, 22.5 kDa), which has 64.8% identity to Ami from strain S16 and 36.7% to NicF from strain KT2440. Finally, maleate cis/trans-isomerase (Iso, 26.8 kDa) catalyzes the isomerization of maleic acid into fumaric acid, which exhibits an identity of 78% to Iso from strain S16 and 71.6% to NicE from strain KT2440. Iso from strain S16 presents a specific activity of 9.4 U/mg and an apparent *K_m_* of 0.69 mM for maleic acid at pH 8.4 and 30°C. The crystal structures of Nfo, Ami, and Iso from strain S16 have been reported, providing a comprehensive understanding of the catalytic mechanisms of these enzymes (Chen et al., 2013; Wu et al., 2014). Finally, the end product fumaric acid enters the classic TCA cycle for further catabolism.

*A. tumefaciens* S33, *Shinella* sp. HZN7, and *Ochrobactrum* sp. SJY1 can grow aerobically with nicotine as the sole source of carbon and nitrogen. They catabolize the alkaloid with the nicotine-degrading enzymes to support their life. Meanwhile, they conserve energy from its oxidation. In connection with the TCA cycle, oxidation of nicotine provides metabolites, reducing power (electrons), and energy (ATP) for anabolism. Among the six oxidoreductive reactions in the hybrid pathway, three are catalyzed by oxidases which transfer electrons from their substrates directly to O_2_ (Figure 2B). The other three reactions deliver electrons to an electron carrier. NdhAB utilizes Paz as its electron acceptor, which can further deliver the electrons to cytochrome *c* and then to the final electron acceptor O_2_ catalyzed by cytochrome *c* oxidase, or directly to O_2_ considering the pseudospecificity between Paz and cytochrome *c* (Yu et al., 2017a). Pno transfers the electron to EtfAB, and then to CoQ catalyzed by Euo, which enters the classic electron transport chain (ETC; Wang et al., 2019a). Ald uses NAD(P) as an electron acceptor, and the reduced equivalent can be reoxidized when it takes part in the reaction of Hsh. Alternately, the reduced equivalent also can be reoxidized by NADH dehydrogenase complex, by which the electrons enter the classic ETC. The routes for electron transport involved in nicotine oxidation *via* the hybrid pathway are thus constructed, and ATP can be further synthesized *via* chemiosmosis.

## Perspectives

The genomic, transcriptomic, and biochemical analyses have revealed the mysterious mechanism of the novel hybrid pathway. To completely understand the molecular mechanism, further studies on the molecular evolution, transcription regulation, substrate sensing and transportation, and detoxification of reactive intermediates are still needed. In addition, it is crucial to resolve the structures of some key enzymes, transcriptional regulators, and chemotaxis proteins to discover their catalytic mechanism and substrate sensing/interaction. This will benefit the development of new technologies, for example, transforming nicotine into valuable chemicals or constructing bio-sensors for detecting nicotine and its metabolites with wild-type or engineered whole cells and enzymes. Recently, whole cells of genetically engineered strain S33 have been used as catalysts to transform nicotine from tobacco wastes to 6-hydroxynicotine (Yu et al., 2017b). An enzymatic route has also been constructed to produce 2,5-dihydroxypyridine (Li et al., 2014). A more extensive investigation of the enzymes will consequently pave the way for their application within industrial biocatalysis.

## Author Contributions

HH and SW designed the minireview. All authors wrote, revised, and approved the manuscript.

### Conflict of Interest

The authors declare that the research was conducted in the absence of any commercial or financial relationships that could be construed as a potential conflict of interest.
